# Supplementary material for: The Role of Epigenetic and Biological Biomarkers in the Diagnosis of Periodontal Disease: A Systematic Review Approach
Source: Diagnostics (Basel). 2022 Apr 7;12(4):919. doi: 10.3390/diagnostics12040919 (PMC9029524; doi:10.3390/diagnostics12040919)
Supplement: Supplementary file 1 [file diagnostics-12-00919-s001.zip › diagnostics-1626437-Supplementary.pdf]

S1

Table S1.1 Displaying the results of the Embase search conducted from 27th September-14th October 2020.

Search Term	Epigenetic	Results	Periodontal Disease	Results	Diagnosis	Results
**MeSH**	Epigenetics	79123	Periodontal Disease	44314	Diagnosis	1339517
**Keywords**	Epigenetic* or epigenomic* or biomarker* or "biological marker*"	672229	Periodont* or gingivitis or periodontitis	113844	Diagnos*	6462420
**MeSH OR Keywords**		672229		113844		6462420
**AND**						894
**Limits Applied**						359

Table S1.2 Displaying the results of the Medline search conducted from 27th September-14th October 2020.

Search Term	Epigenetic	Results	Periodontal Disease	Results	Diagnosis	Results
**MeSH**	Epigenomics	5733	Periodontal Diseases	26162	Diagnosis	17371
**Keywords**	Epigenetic* or epigenomic* or biomarker* or "biological marker*"	678836	Periodont* or gingivitis or periodontitis	103576	Diagnos*	5060776
**MeSH OR Keywords**		678836		103576		5060776
**AND**						823
**Limits Applied**						303

Table S1.3 Displaying the results of the Dentistry and Oral Sciences Database search conducted from 27th Septebmber-14th October 2020.

Search Term	Epigenetic	Results	Periodontal Disease	Results	Diagnosis	Results
**Keywords**	Epigenetic* or epigenomic* or biomarker* or "biological marker*"	3249	Periodont* or gingivitis or periodontitis	68665	Diagnos*	56884
**AND**						412
**Limits Applied**						167

Table S1.4 Displaying the results of the CINAHL search conducted from 27th September-14th October 2020.

Search Term	Epigenetic	Results	Periodontal Disease	Results	Diagnosis	Results
**Subject Heading**	Epigenomics	1395	Periodontal Diseases+	20465	Diagnosis+	1877811
**Keywords**	Epigenetic* or epigenomic* or biomarker* or "biological marker*"	96908	Periodont* or gingivitis or periodontitis	24296	Diagnos*	1071234
**Subject Heading OR Keywords**		96908		28373		2293215
**AND**						542
**Limits Applied**						180

S 2

Table S2.1 Illustrating Fold Change (FC) and Variability Importance Projection (VIP) of the metabolites analyzed in the study by Chen *et al.*

Decreased in Disease	Metabolite	FC	VIP
	2-Ketobutyric Acid	0.06	1.74
	Glycine-d5	0.08	1.14
	Thymidine	0.08	1.71
**Increased in Disease**			
	Noradrenaline	30.29	1.71
	Ribose	21.22	1.78
	Dehydroascorbic Acid	18.62	2.14
	Lysine	13.75	1.10
	Xanthine	13.66	1.09

Table S2.2 Illustrating the Fold Change (FC) and Variability Importance Projection (VIP) of the metabolites analyzed in the study by Pei *et al*.

**Decreased in Disease**	**Metabolite**	**FC**	**VIP**
	Lactamide	0.65	1.76
	O-Phosphoserine 1	0.71	1.00
	1-Manopalmitin	0.72	1.16
**Increased in Disease**			
	Glycine-d5	20.38	2.69
	N-Carbamylgutamate	9.83	2.63
	Fructose-1	5.92	2.39

Table S2.3 Illustrating the False Discovery Rate (FDR) of the metabolites analyzed in both Generalized Chronic Periodontitis (GCP) and Generalized Aggressive Periodontitis (GAgP) in the study by Romano *et al.*

		FDR	
**Decreased in Disease**	**Metabolite**	**GCP**	**GAgP**
	Pyruvate	0.0001	0.0044
	N-Acetly Groups	0.0086	0.0481
	Lactate	0.0007	0.0044
**Increased in Disease**			
	Proline	0.0221	N/A
	Phenylalanine	0.0007	0.0099
	Isoleucine	0.0029	N/A
	Valine	0.0037	N/A
	Tyrosine	0.0297	0.0481
	Formate	N/A	0.0086

Table S2.4 llustrating the Odds Ratio of metabolites between periodontal health and disease in the study by Rzeznik *et al.*

Decreased in Disease	Metabolite	OR
	GABA	33.48
	Lactate	13.46
**Increased in Disease**		
	1-Butyrate	14.82

Table S2.5 Illustrating the Positive Predictive Value (PPV), Negative Predictive Value (NPV), Sensitivity and Specificity of the metabolites outlined in Table 2.4.

**PPV**	**NPV**	**Sensitivity**	**Specificity**
0.77	0.86	0.89	0.72
